# Complexities and dilemmas in community consultation on the design of a research project logo in Malawi

**DOI:** 10.1371/journal.pone.0205737

**Published:** 2018-10-18

**Authors:** Deborah Nyirenda, Kate Gooding, Wezzie Lora, Moses Kumwenda, Meredith McMorrow, Dean Everett, Nicola Desmond

**Affiliations:** 1 Malawi Liverpool Wellcome Trust Clinical Research Programme, Chichiri, Blantyre, Malawi; 2 University of Malawi, College of Medicine, Chichiri, Blantyre, Malawi; 3 Liverpool School of Tropical Medicine, Liverpool, United Kingdom; 4 Influenza Program, U.S. Centers for Disease Control and Prevention-South Africa, Pretoria, South Africa; 5 U.S. Public Health Service, Rockville, Maryland, United States of America; 6 The Queens Medical Research Institute, University of Edinburgh, Little France Crescent, Edinburgh, United Kingdom; University of Toronto, CANADA

## Abstract

**Background:**

Community engagement on research design is widely highlighted as an important approach for ethical research. This article reports the experience of consulting with communities on the logo used for an influenza study in Malawi. The logo was designed for use on badges worn by study researchers, participant information sheets and other project documents, and could affect perceptions of the study and consequent engagement in the research.

**Methods:**

Four focus group discussions were conducted with populations targeted by the influenza study: pregnant women, people with HIV, mothers and community members. The focus groups incorporated a participatory matrix exercise focusing on key themes emerging from the discussions such as: attractiveness, comprehension, acceptability and suggestions for improvement. Findings from the focus groups were analyzed according to these key themes.

**Results:**

The consultation highlighted important benefits of discussion with communities on research design, including providing new perspectives and helping to avoid harm. For example, people living with HIV felt that one of the possible logos could increase stigma within communities. The experience also indicated potential challenges of consultation. In particular, there were contrasting perspectives among the groups, such that the consultation did not provide a clear answer about which logo should be selected.

**Conclusions:**

Our experience adds to current evidence on community engagement by reporting on an area where there is less discussion of community consultation for design of a study logo. The consultation exercise reaffirmed the value of community engagement, but also the difficulty of relying on a brief consultation for decision-making in research design. Further ethical guidance is required on how to negotiate contradictory views during consultations.

## Background

Community or public engagement is increasingly recognized as a critical component of international medical research [1,2]. Guidelines and existing literature highlight the need for active community engagement at different stages of research, and the value of engagement for both ethical and effective research practice. Engaging communities and incorporating their distinctive values, culture and social practices in study design and implementation demonstrates respect for potential participants, improves trust, and increases participation and long-term engagement in the research [1,3].

While public/patient involvement in research planning is widely recommended [4,5], a study logo may be seen as a relatively trivial aspect of research design that does not require community consultation. However, evidence from social psychology indicates the importance of branding, and in particular the strength of visual images [6]. The need to develop appropriate logos is widely recognised in commercial marketing: an effective logo builds positive consumer perceptions and trust in the company and its products [6,7]. The same may apply in health research. Evidence suggests that community decisions on engagement can depend on their perceptions of the research institution more than their knowledge of study aims [8,9]. Study logos are an important part of institutional identity and image, and as such logos can affect decisions around participation. For example, in Kenya images of snakes in a study logo led to negative rumours about the research, concerns among communities and a lack of trust in both the study and the research institution [10]. For community members, the snakes depicted in the logos symbolised devil worship, a belief strengthened by the prominence of drawing blood as one study activity. In the UK, researchers for a cohort study among young people with cancer found that involving potential participants in study branding, including logo selection, appeared to increase acceptability and improved both participation and retention [6].

Given the importance of study logos for community perceptions and engagement, we sought feedback from potential research participants on the logo for a programme of influenza research in Malawi. Seasonal influenza epidemics are estimated to cause 250,000–500,000 deaths each year worldwide [11]. Difficulty in identifying influenza and limited surveillance systems mean the extent of influenza in sub-Saharan Africa is not fully known. However, available data suggest influenza causes significant morbidity and mortality in the region, particularly amongst young children and people with HIV [12,13]. Surveillance data from 15 African countries found that 10% of severe acute respiratory infections were associated with influenza viruses [14]. An estimated 99% of deaths from influenza-associated acute lower respiratory infection in children under five occur in developing countries [15]. Annual influenza vaccination is recommended for preventing influenza virus infection and its complications [16] and the World Health Organization's Strategic Advisory Group of Experts recommends influenza vaccination for children under 5 years, the elderly, pregnant women, individuals with high-risk underlying conditions and healthcare workers [17]. Assessment and introduction of vaccination requires further evidence on the extent of influenza and effectiveness of vaccination in African countries, particularly in populations with high rates of HIV, malaria and malnutrition. To improve the evidence base for potential introduction of an influenza vaccine, the Malawi-Liverpool-Wellcome Trust Clinical Research Programme (MLW) is undertaking a programme of studies on influenza through support from a CDC Influenza Division Research co-operative agreement. This includes research to examine the severity and extent of influenza among high risk groups, including children, pregnant mothers and HIV-infected adults, the effectiveness of an influenza vaccine in children living in malaria endemic areas, and the acceptability of influenza vaccination. This research programme includes work in urban Blantyre, including hospital-based surveillance studies, and in rural Chikhwawa District.

The influenza research team felt that a logo would improve public recognition of the influenza studies and support community participation. The logo had several rationales: it was designed to make the research visible, to encourage people to ask about the studies, and to prompt conversations about influenza. The logo was to be used on badges worn by study researchers and fieldworkers, and on participant information sheets and other project documents. Given the need to ensure the logo was socially and culturally appropriate, we engaged potential study participants in the logo development.

This paper contributes to a growing body of literature that examines community engagement in practice by documenting our experience of the consultation exercise. The consultation highlighted benefits of public consultation but also complexities, including the difficulty of identifying clear answers through discussions with diverse stakeholders. While consultation was designed to support community input and ensure the logo was socially and culturally acceptable, the exercise also raised questions about the feasibility, effectiveness and legitimacy of the consultation approach. We reflect on the benefits and challenges to identify lessons for other research projects.

## Methodology

### Setting

Malawi is a land locked country located in Southern Africa with a population of 17,215,000 [18]. The majority of the population (84%) reside in rural areas, and 72% of the population live below the poverty line on less than US$1.25 a day [18]. The country has a heavy disease burden due to both infectious and non-communicable diseases, such as HIV/AIDS, malaria, pneumonia, diarrhoea and TB [19–21]. Relative to population, Malawi produces high numbers of research outputs through both local and international institutions [22]. However, there is limited understanding of health research among the general public, with widespread misconceptions about the risks and benefits of participation in research [23,24]. For example, participants may enroll due to therapeutic misconception, believing they will gain access to better health services [24–26]. Alternatively, concerns about drawing blood, fear of strangers and perceived lack of benefit from the study can reduce participation [23]. These community perceptions and understandings affect implementation of research, and heighten the importance of community consultation on research procedures.

The Focus Group Discussions (FGDs) were conducted with participants from both urban and rural settings in Blantyre and Chikhwawa districts. Bangwe and Ndirande are high-density urban areas in Blantyre. Both areas have high levels of in and out migration, and they are low-income with a variety of formal and informal income earning strategies. Mfera is a rural village in Chikhwawa with high levels of poverty. Both Blantyre and Chikhwawa are long-term research sites for MLW, and each district hosts a range of trials, observational and qualitative studies.

MLW undertakes community engagement activities in all these areas, including use of radio programmes, media engagement, science cafes, community film shows, a science exhibition and representation from community advisory groups. The consultation about the logo is part of a wider programme of ongoing public and community engagement around the influenza programme and MLW’s research more generally. For the influenza programme, engagement includes collaboration with community advisory groups for feedback on study procedures. In addition, meetings are held throughout the research process with community leaders, district health staff and community members. This is done to explain the research, address community concerns and share preliminary findings.

This small consultation exercise was part of the community engagement activities to ensure that the study logo was culturally and socially appropriate, and hence we did not seek ethics approval. Ethical guidelines on community engagement state that *‘examples of community engagement processes that may require ethics review include systematic data collection that can be generalized and disseminated in forums outside of the community in which they were implemented*, *as well as any data generation that could create social risks for participants’* [27]. This consultation exercise was undertaken to seek feedback on the logo rather than generalizable data, and we anticipated minimal social risks among FGD participants. Study information was discussed with FGD participants and verbal consent was sought prior to FGDs. Focus group participants were also told during the consenting process that the group discussions were going to be shared with other researchers or published. In addition, we presented this paper to MLW Community Advisory Group (CAG) members to seek their views on whether it was acceptable to publish this work. The CAG members who provide feedback to represent community views on MLW research gave consent to publish the work since this was part of the formative work and not a research with human subjects.

### Consultation aims and design

**Fig 1:**
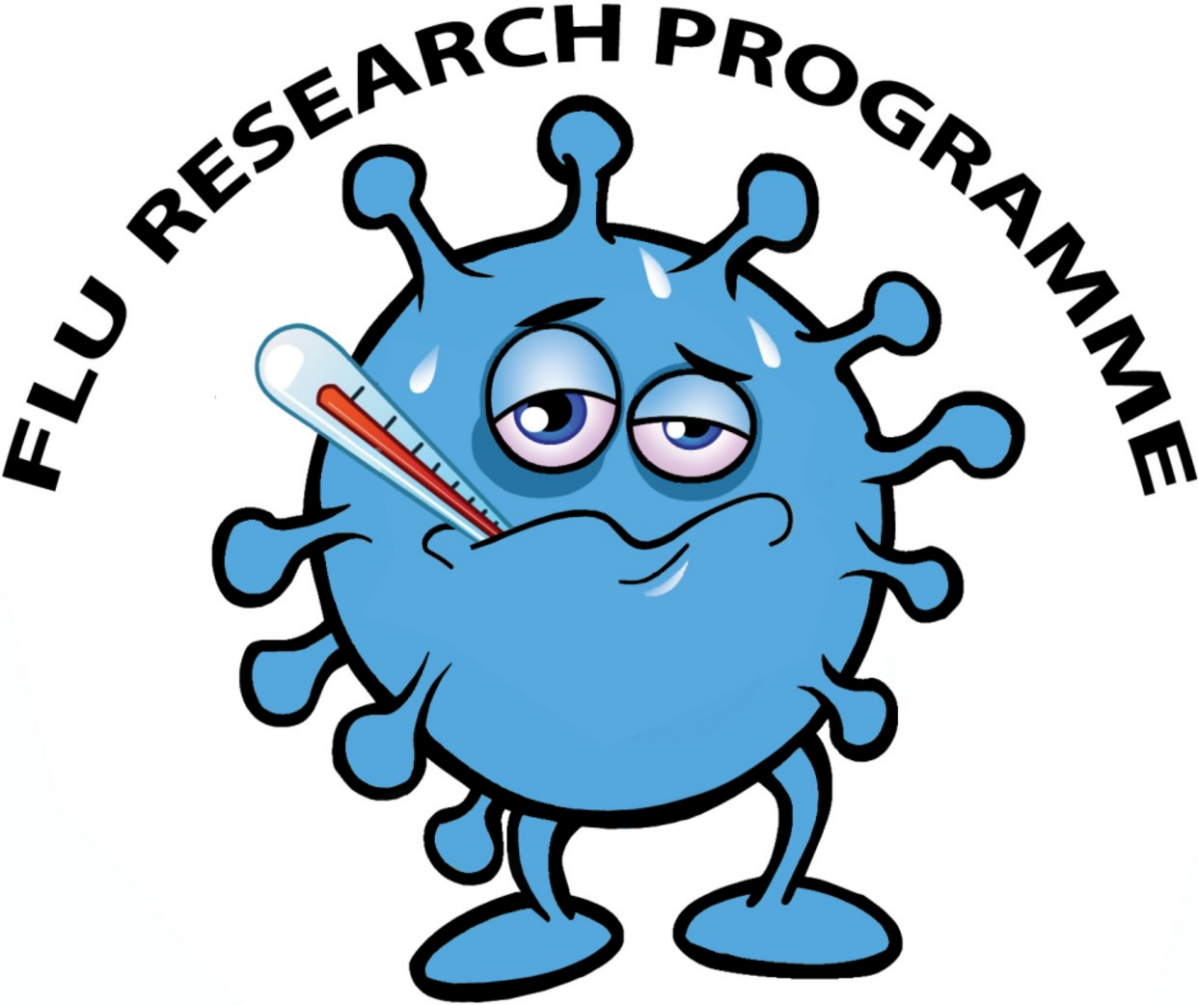
Cartoon—Logo A.

**Fig 2:**
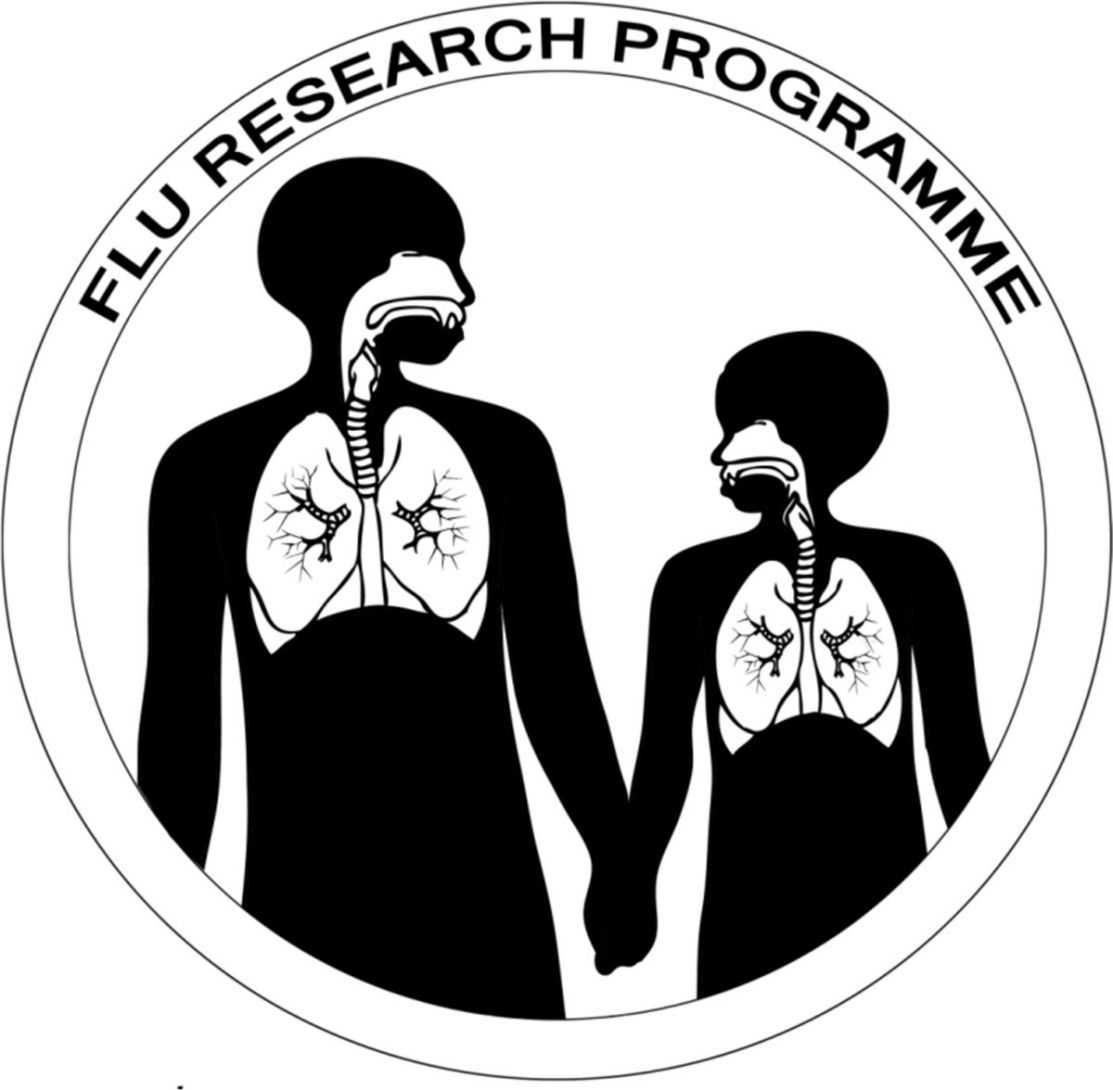
Human chest cavity- Logo B.

The consultation aimed to support the choice of logo for the flu studies. Two potential logos were developed by the study team with support from a professional artist. Logo A was a cartoon representing a virus that causes flu, and Logo B portrayed the internal structure of the human respiratory system (see Figs 1 and 2). The consultation sought to examine views among potential study participants about whether they found the logos understandable and acceptable, and to identify which of the two logos would be more appropriate. By engaging potential study participants and exploring their views about the two logos, we aimed to ensure that logo selection was based on evidence about community preferences.

## Methods

We used four FGDs with community members in the influenza study catchment areas. The team decided to use focus groups because of their value in identifying social norms and facilitating an understanding of standard and acceptable behavior in communities. Two focus groups were conducted in urban Blantyre, one with people living with HIV from Bangwe Health Centre, and one with pregnant women attending an antenatal clinic at Zingwangwa Health Centre. Two further focus groups were conducted in Chikhwawa District, one with mothers of under-five children attending Mfera Health Centre and one with people from the villages surrounding Mfera Health Centre (who will be referred to as community members in this paper). Details of participants’ characteristics are presented in Table 1.

**Table 1:** Socio demographic details of focus group participants.

	FGD 1: Pregnant women (n = 7)	FGD 2: People living with HIV (n = 10)	FGD 3: Mothers (n = 8)	FGD 4: Community members (n = 10)	Total
Age (years)	** **	** **	** **	** **	
15–20	1	2	1	1	5
21–25	1	0	4	1	6
26–30	3	0	3	1	7
31–35	1	2		4	7
36–40	1	3		1	5
>40		3		2	5
					
Education					
None		1			1
Primary	5	3	7	8	23
Secondary	2	6	1	2	11
					
Sex					
Female	7	5	8	3	23
Male		5		7	12

This selection of participants was based on the groups targeted by the influenza studies: research on severity of influenza among vulnerable groups sought to recruit people with HIV and pregnant women in Blantyre, while the vaccine trial took place in Chikhwawa and sought to recruit children under five, and consequently required engagement of their parents (primarily mothers as the main caregivers in Malawi). The community focus group was designed to indicate wider perceptions among a more diverse range of people.

The staff in charge of each of the three health centres were contacted to discuss the consultation exercise, in order to arrange access to participants. On the day of the focus groups, healthcare workers at the centres used convenience sampling to select participants attending the health centres that day. Criteria for selection for the first three groups were that participants should be members of the target group (people attending ART clinics, pregnant women or mothers of under-five children), and willing and available to participate in the focus groups. We also asked a staff member to help recruit participants for the fourth focus group, which included men, women, boys and girls from the village surrounding Mfera Health Centre.

### Data collection

A topic guide was used to facilitate the focus group discussions, and a participatory ranking exercise was incorporated for participants to assess the logos. The topic guides included questions on whether participants liked the logos, the perceived meaning of the logos, whether they would be understood and accepted in communities, and suggestions for a Chichewa name for the logos. An opportunity was also given to the participants to discuss their general views in order to allow them to cite inductive ideas. The ranking exercise was designed to provide quantitative information on acceptability and a clearer indication of variation in preferences across different groups of potential research participants in order to inform decisions. During the focus group discussions, posters of the two logos were placed in the front of the room and participants were led through a discussion about their perception of the logos. Attributes discussed by participants were listed on a flip chart, and used as criteria for assessing the logos during the ranking exercise. These were later grouped into three categories of attractiveness, comprehension and acceptability. The latter was tested by asking participants if they would wear a t-shirt with the logo. Each participant was then asked to put a colored sticker on the attributes that they mostly identified with about each logo. This was followed by another discussion on the rationale behind their ranking. Each focus group was facilitated by experienced social scientists (DN, WL, MK), and these three social scientists also took turns to take notes during the discussions. The FGD lasted one hour and were recorded using a digital recorder. FGD participants were given a refreshment and they were also compensated for their time.

### Data analysis

Two social scientists (DN and WL) listened to recordings from the FGD separately and developed a coding framework. The recordings were audio coded and emerging codes were grouped into analytical themes. A framework matrix was developed based on these themes and populated with relevant notes and discussions about each logo. The initial themes and matrix were later reviewed by other researchers (MK and KG). Findings were analyzed by comparing responses across different groups and in relation to sex and location. Scores from the matrix ranking exercises were also used to support discussions on main themes of attractiveness, comprehension and acceptability. Verbatim quotes have been used to illustrate key themes.

## Findings

### Participants’ perspectives

In this section, we report the findings around the primary issues considered during analysis: the attractiveness of the logo, comprehension, acceptability, and suggestions for improvements and Chichewa names. We structure the findings around these categories because they were the basis for the FGD structure and matrix scoring, but issues raised by participants during the discussions sometimes relate to multiple themes. We combine insights from the discussion and the focus group matrix scores, which are shown in Table 2. Within the table, a higher score indicates a more positive rating.

**Table 2:** Number of focus group participants preferring Logo A vs. Logo B.

Theme	Logo A (Cartoon logo)					Logo B (Chest cavity logo)				
	FGD 1	FGD 2	FGD 3	FGD 4	Total	FGD 1	FGD 2	FGD 3	FGD 4	Total
Total participants	7	10	8	10		7	10	8	10	
Attractiveness	0	10	7	4	**21**	7	0	1	6	**14**
Comprehension	0	10	5	0	**15**	7	0	3	10	**20**
Acceptability	0	10	4	1	**15**	7	0	4	9	**20**
Total					**51**					**54**

Key

FGD 1 Pregnant women; FGD 2 People living with HIV; FGD 3 Mothers

FGD 4 Community members

#### Attractiveness of the logos

We defined attractiveness as whether people liked the logo. We asked participants what they liked and did not like about each logo, and which logo they would prefer to see. The matrix scores indicated mixed views between and within the groups: people with HIV voted for Logo A as more attractive, a view shared by the majority of mothers. The group of pregnant women, however, voted for Logo B as more attractive, and votes of the community members were split between Logos A and B. Variations in voting and preferences did not appear to be related to characteristics of the FGD participants such as age, gender, education level or location. For instance, a majority of participants in two FGDs were women below the age of 30 (FGD 1 with pregnant women and FGD 3 with mothers) while the other two FGDs with people living with HIV (FGD 2) and community members (FGD 4) were conducted with a mix of both men and women.

Through the group discussions, three key issues emerged that affected attractiveness. One aspect mentioned by participants was color. Most participants in the groups that voted in favor of Logo A (people with HIV and mothers) reported that they preferred the blue color of Logo A, whereas Logo B was just black and white. However, there were also positive comments in favor of the black color of Logo B from some participants in the people with HIV group, so this preference was not consistent. In addition, although participants in the pregnant women focus group all voted for Logo B as more attractive, some of these participants also expressed negative comments about its black color.

Another aspect that affected the logos’ perceived attractiveness was how abstract or how clear the health messages were. Most participants did not initially know what Logo A represented, and they felt that *“most people would not know what it is”* (FGD 2). This initial lack of comprehension was seen as a positive characteristic by some groups: participants across the groups felt Logo A would raise curiosity and stimulate people’s interest to learn more about what the logo represented. However, some participants in FGD 1 (pregnant women) and FGD 4 (community members) preferred the more literal design of Logo B, because they felt it indicated a positive health message. For them, Logo B was self-explanatory and indicated the importance of seeking proper diagnosis and treatment.

The third aspect of attractiveness related to potential stigma, and it was affected by logo abstractness. Participants in FGD 2 with people living with HIV felt Logo B would be associated with tuberculosis because it depicted a chest cavity, and consequently that it may create stigma for those involved in the research. The more abstract design of Logo A was seen as avoiding this risk:

*“If we choose the cartoon we will be concealing ourselves*. *To say the truth*, *many of us hide our HIV status… So*, *we can choose the cartoon because people will just say that it is a toy (Logo A) … This one is showing exchange of breath*, *so people will be saying that it is about TB*. *This picture can put me in problems because people will be asking questions just to mock me and not in good faith*.*”* **Man, FGD with people living with HIV**

#### Comprehension of the logo meaning

Comprehension was defined as the ability of participants to understand the message portrayed by the logos, as defined by the flu study team. We sought to understand how participants interpreted the logos, and whether the logos portrayed sufficient information about the research. At the start of the group discussions, before researchers explained what the logos were supposed to represent, we asked participants what they saw in the logos, what they thought the logos meant and what message the logo was trying to communicate.

In terms of the matrix scores, all participants in FGD 2 (people living with HIV) and five of the eight participants in FGD 3 (mothers) voted for Logo A as more easily understood, whereas all participants in FGDs 1 (pregnant women) and 4 (community members) voted for Logo B as more comprehensible. As indicated under attractiveness, most participants could not understand Logo A at first sight. This was indicated in varied ideas about what Logo A represented, including a *head with legs*, *big eyes and horns; sunshine; a crab with eyes and legs; a frog; a tortoise; a cartoon;* and *a doll;* as well as comments that “*we do not know what it is”*. These misunderstandings also came from some participants in the groups that voted for Logo A as easier to comprehend, some of whom initially thought Logo A was a '*balloon'* or a '*toy'*. However, some participants did relate the cartoon more closely to health, describing the logo as *'a germ that causes malaria*, *Ebola and other diseases'*. These comments came from a small number of participants in the group that voted for Logo A as comprehensible (FGD 2, people living with HIV), as well as participants from FGD 4 (community members), none of whom voted for Logo A as more comprehensible.

In contrast, most participants understood Logo B as showing the lungs and chest cavity, probably because the logo is similar to pictures with which they were familiar from hospitals or schools. The link to influenza was not made strongly for either logo until the designs were explained, but some felt the link with influenza was clearer with Logo B:

*“This one portrays a lot of lessons*, *if a person reads the message and sees the picture they will be able to tell that a person who is suffering from flu looks like this (Logo B)*. *It is difficult for people to tell if indeed that picture (Logo A) shows a person*, *yes we can see that it looks like a bug but they won't understand the connection between the bug and the disease it causes*.*”* **Woman, FGD with pregnant women**

However, Logo B was not always interpreted in the way intended by the researchers. For example, some participants perceived the white color in the chest cavity as an illustration of infections (rather than just the lungs), while participants in the group of people living with HIV thought it illustrated breast enlargement among men as a side effect of antiretroviral therapy (ART), indicating the importance of individual context on interpretations.

While most participants initially felt Logo B was easier to comprehend, some participants switched their preference after the facilitators explained the intended meaning of the logos, instead preferring Logo A. This pattern was notable in the groups of people living with HIV, mothers and community members. Although they felt the logo would still not be initially understood without prior knowledge, they felt this had positive implications: lack of initial understanding could spark curiosity as indicated under attractiveness, and could have value for health education because people might learn more about the causes of influenza through the logo. On the latter, some participants stated that influenza is generally not considered a disease in their communities, so learning that a bug (as depicted on Logo A) causes influenza would be new information:

*“Most of us didn’t know that there is a bug that causes flu*. *When we get flu we think that ‘eeh maybe it is malaria’*. *Most of us didn’t know it*, *so our eyes have been opened now that aah*! *This is the bug that causes flu*. *We just thought that flu is not a disease…people want to see that there is indeed a bug that causes this disease*.*”* **Man, FGD with community members**

#### Acceptability of the logo

We defined acceptability as whether the logos’ appearance and message satisfied community standards, norms and values and whether the logo could be incorporated into communities’ daily lives. To assess acceptability, we asked participants whether they would wear t-shirts with the logos, whether there was anything about the logos that communities would find offensive or inappropriate, which logo people would find culturally acceptable, and how people would react if they found the logos at the clinic.

As with attractiveness and comprehension, there were mixed views about acceptability, due to both appearance and understanding of the logos. In terms of the matrix scores, all the people living with HIV and most mothers voted in favor of Logo A, whereas all the pregnant women and a slight majority of community members voted for Logo B. Aspects of acceptability raised in the discussions overlapped with issues raised in relation to attractiveness and comprehension. The abstract nature of Logo A again emerged as an area of mixed views. Asked about likely community reactions if they were to wear a t-shirt with Logo A, several participants felt people would ask lots of questions. Some saw this as a positive effect linked to raising curiosity, but others thought inability to understand the logo would mean people might think it was just *'useless'*, and some preferred a more self-explanatory t-shirt:

*“People won't ask you any questions about this T-shirt*, *they will be able to tell for themselves (Logo B)*, *but you will get a lot of questions from that one (Logo A*.*)”* **Woman, FGD with mothers**

Stigma was also raised again, with concerns about a t-shirt portraying Logo B among FGD 2 participants (people living with HIV), who associated Logo B with HIV and TB stigma. However, while some people had concerns, others felt both logos were acceptable. Overall each logo had benefits and drawbacks. Logo A was acceptable because it sparked interest and avoided stigma, but Logo B was more acceptable because it was self explanatory.

### Suggestions for improvements and Chichewa names for the logo

Participants were asked to suggest how the research team could improve the design of the two logos to make them more attractive, understandable and acceptable. Comments from participants mainly related to making the logos easier to understand. In regards to Logo A, some participants in FGD 1 (pregnant women) suggested that researchers should develop a logo of the real influenza virus, not the cartoon in the version presented. Participants in FGD 4 (community members) suggested that the thermometer should be removed from the cartoon, on the basis that viruses do not have thermometers in their bodies. In regards to Logo B, many participants in FGD 2 (people living with HIV) and FGD 3 (mothers) suggested that the logo should incorporate the influenza bug, so that people realize the logo relates to influenza research:

*“In that picture*, *they should put the flu bug going into the body*. *In that case we will be able to know that this is about flu research*.*”* **Woman, FGD with people living with HIV**

We also asked participants to provide us with Chichewa names for each logo. The majority of suggestions for Logo A were names suggesting something that causes disease, such as *“chilombo” (bug)*, or *“akita” (actor)*, which meant the *culprit*, because the virus is the culprit for influenza infection. Others however suggested that the word *Chimfine (flu*) should be added to the logo in order for people to understand it. Participants also suggested names that represent risk, such as *“tseka mphuno” (close your nose)*, or *“chenjerani”*, which means *beware*. In contrast, names suggested for Logo B were not related to the influenza virus or to research. For example, *“yanjanani”* (get together) and *“chikondi”* (love), because Logo B has a picture of two people who seemed to represent a couple. This raises again the issue of comprehension of the logos: by the end of the FGDs, the link to influenza research may have been clearer with Logo A. Interpretations of Logo B as a couple may also have contributed to its association with HIV and consequent concerns about stigma.

In the absence of a clear participant preference, the research team decided to vote among themselves on which of the two logos to use. Initially the entire influenza team voted and their vote was roughly split. Given the need to make a decision to move the studies forward, the lead investigator then had a casting vote, and decided in favor of Logo A. This decision considered in particular the risk of stigmatization from Logo B raised by people with HIV, and the value of Logo A in prompting conversations and questions about influenza and the research. Although the consultation did not provide a definite answer, it did provide researchers with knowledge to place more weight on perceptions that could minimize harm to potential study participants.

## Discussion

The consultation enhanced our understanding of how community members with diverse backgrounds, levels of education and knowledge interpreted and reacted to the logos. The exercise provided valuable information that supported selection of the study logo. A particular benefit was highlighting potential risks related to Logo B: the concerns among people living with HIV about stigma indicated risks that were unforeseen by the study team, and were a significant factor behind the final selection of Logo A. Without public consultation, we might have selected a logo that not only discouraged engagement but caused harm for some participants. In this respect, our experience supports the value of public consultation for the ethical goal of enhancing protection, by indicating risks not apparent to researchers [28].

The exercise also highlighted complexities and challenges of consultation. Perhaps naively, we anticipated that the focus groups would provide a clear answer about which logo was preferred by potential participants. However, we were left with a picture of diverse preferences, and with uncertainty about whether expressed views reflected views of potential participants in practice. One issue was that the discussions showed a lack of consensus between and within groups about which logo to use as indicated in the matrix voting. In some cases, directly contradictory views were also expressed by participants in the same FGD. For example, some pregnant women stated that Logo A was easy to understand, while others thought that people would not understand it. Ethical guidelines state that *‘disagreements regarding the design and conduct of the research must be subject to negotiation between researchers and community leaders’* [27]. There is however a lack of clear guidance on how to negotiate contradictory views of community partners, even though ethical guidelines recommend that consultation processes must ensure a diversity of views. Our findings suggest a need for ethical guidance on how research teams ought to negotiate contradictory views among community partners during consultation processes.

Diverse and potentially opposing views among communities are noted within the community engagement literature [29]. These contrasting views among potential participants raise the dilemma of whose views should be incorporated in research decisions. Assessing group preferences was complicated by differences between the voting and opinions suggested in the discussion. In some cases, the scores suggested a unanimous group preference for one logo, but the discussion did not indicate any strong group preference about each logo. For example, participants in FGD 1 (pregnant women) all voted for Logo B as more attractive, but they also expressed negative comments in the discussion about its attractiveness. This may be because while one logo was preferred, there were still reservations about its attractiveness, such that the need to choose means the matrix scores gave an incomplete understanding of group preferences. In addition, the unanimous scores on all criteria for FGD 1 and 2 may have reflected peer influence and a decision to vote in the same way as other group members, an inherent feature of FGD that we return to below.

A further issue muddling the picture of participant preferences is that opinions can change, and participants’ views shifted during the discussions in response to issues raised by other group members, and as they received more information from the researchers about the logos. Some participants initially preferred a logo with the chest cavity because it was familiar, but once the meaning of the bug logo was explained, some changed their opinions and voted for Logo A. This suggests that perceptions might be affected by the presentation of the study and sensitization on the meaning of the logo. Consequently, a negative response might not rule out a particular logo, but rather suggest that it needs to be explained to communities carefully.

The changing views during discussions and unanimous voting in some FGDs both raise the issue of social influences within focus groups. FGDs were ideal because they tend to indicate social norms and perceptions based on social interaction [30]. Previous research shows that decisions on research participation often respond to information gained through social networks and local community interactions [31,32]; there was value in examining decision-making within this social context. However, the group approach may also have had the disadvantage of obscuring individual views and responses to the logos. Although the matrix ranking exercise was designed to indicate individual views, the tendency for groups to vote the same way suggests the individual votes were influenced by group discussions. This could have reflected the presence of dominant personalities within the groups. Some groups included participants with different levels of social status and influence (for example, men, women and youths), and this may have encouraged a tendency to follow the views of socially respected participants. This could have been partially avoided through a more homogeneous selection of focus group participants.

While peer pressure may have been one factor in changing views during the FGD, this also reflects the inherently social nature of knowledge and perceptions, with views on acceptability developing in response to ideas from other people [33]. Participants may have shifted their positions as discussions progressed because they were persuaded by other people's rationales, rather than because of pressure to conform. In this respect, while shifting views complicate the assessment of acceptability from group discussions, they perhaps present a more realistic reflection of the nature of acceptability than a static and uniform indication of preferences.

In addition to the varied and changing views, the findings also raised a question for researchers about the weight to place on participants’ comments. Some suggestions for improvement from participants were based on their assumptions about what would be understood or accepted by other community members. For example, one participant suggested that showing the bug going into the body would clearly indicate that the research is about flu. This assumption and perception may not be a reflection of reality because people do not see a virus going into the body as specifically about flu. In addition, FGD participants also suggested Chichewa names that we thought could be potentially confusing to people such as *“chilombo”* (which could either mean bug or animal), *“akita”* (actor), *“tseka mphuno”* (close your nose), and *“chenjerani”* (beware). Decisions about which comments to dismiss and which to take more seriously rested partly on researcher judgment based on the frequency with which issues were raised as well as ethical principle to minimize risks to potential participants. In light of these dilemmas around which comments to accept as important or dismiss; we suggest that the ultimate decision by researchers should be based on ethical principles such as to minimize harm to potential participants.

These mixed findings meant that the consultation did not provide a clear answer about which logo to select. An initial response from the study team was to try to develop an alternative logo that might be more universally preferred among potential participants. An artist was engaged to develop five other logos for further consultations with communities. However, development of these additional logos was a lengthy process, and none of those logos were liked by the study team. Consequently, the team decided to opt for one of the original two logos.

We decided to undertake the consultation because we assumed from previous literature that the logo may influence perceptions. Although the logo was used on t-shirts, badges and study information sheets, the actual effect of the logo on study recruitment and community perceptions was hard to assess. In informal conversations, study staff reported that no participants or potential participants had commented on the logo. A qualitative study linked to one of the trials asked those invited to enroll about their decisions, and the logo was not mentioned in any interviews (unpublished data). It may be that the logo was noticed but was not mentioned because it did not have negative connotations or cause any harm; in this case, the consultation may have had value in helping us choose a logo that avoided harm. However, we think it is also likely that the logo was not sufficiently visible to influence community perceptions. The evidence of a negative effect of study logos from Kenya [10] comes from a situation where the logo is a long-term feature of the research institute and perhaps more visible. The Kenya logo was perhaps controversial, given the traditional connotations of the snake.

The use of a consultation to assess community preferences may have been particularly difficult because of the nature of the questions being asked. In our experience of similar consultation exercises in other studies, eliciting and acting on community feedback has appeared more straightforward. Pictorial diaries have been used in two recent studies, and in both cases community feedback was sought on draft versions of the diaries to see whether the pictures were clear. We can suggest some possible reasons why previous consultation exercises were more effective than the one reported in this manuscript. The flu logo study asked participants to choose between two logos, and with this kind of binary choice, different views among participants are likely. Although varied opinions among participants may exist in a group discussion, varied opinions do not prevent an answer being found. If most participants do understand a picture but a small number do not, investigators should consider making changes so that the picture is clear to everyone.

We intended that the focus group discussions should be a genuine consultation exercise that would allow the research team to follow community suggestions. Guidelines on community engagement emphasize the importance of acting on community views [1,34]. However, the value of consultation, including the benefit for community members as well as researchers, was perhaps less than anticipated. The lack of clear consensus has in some ways made the outcome less responsive to community perceptions than we intended, due to the impossibility of choosing a logo that matched all group preferences and the final decision resting with researchers.

Our timescales and resources limited the consultation process to four FGDs. We were also unable to feedback the findings to FGD participants due to logistical challenges of getting participants who were mostly clinic attendees in one place. We however presented our report to MLW CAG members to get their feedback and consent to publish this paper. A more extensive consultation process may be more effective to generate a consensus between participants and researchers, with more two-way interaction between researchers and participants and more participant input to logo design. For example, communities could be asked to suggest initial ideas for the logo or to comment on revised versions, and different stakeholder groups could be brought together to discuss the initial focus group findings and seek a consensus. This more extensive process could help move community engagement from consultation towards collaboration [34].

## Conclusion

Our experience with the focus groups underlines the value of involving potential research participants in the design of study logos and other research materials, to ensure that these materials are culturally and contextually appropriate. The consultation highlighted important risks of the logos, and allowed the researchers to make a decision based on the ethical principle of minimizing harm to potential participants. Our experience also points to challenges of consultation, in particular the complexities of seeking feedback from multiple groups of potential study participants and negotiating these differing views to reach consensus. As we found, consultations can provide ambiguous or contradictory answers. As such, ethical guidelines on community engagement need to provide further guidance on how to negotiate contradictory views of community partners, in order to make final decisions.
